# Supplementary material for: The interactome of intact mitochondria by cross-linking mass spectrometry provides evidence for coexisting respiratory supercomplexes
Source: Mol Cell Proteomics. 2017 Dec 8;17(2):216–32. doi: 10.1074/mcp.RA117.000470 (PMC5795388; doi:10.1074/mcp.RA117.000470)
Supplement: Supplemental Data [file supp_RA117.000470_133922_0_supp_23978_fzffwf.zip › spectra_annotation/mito_spectra_annotation/87-1-3-1-3-1.pdf]

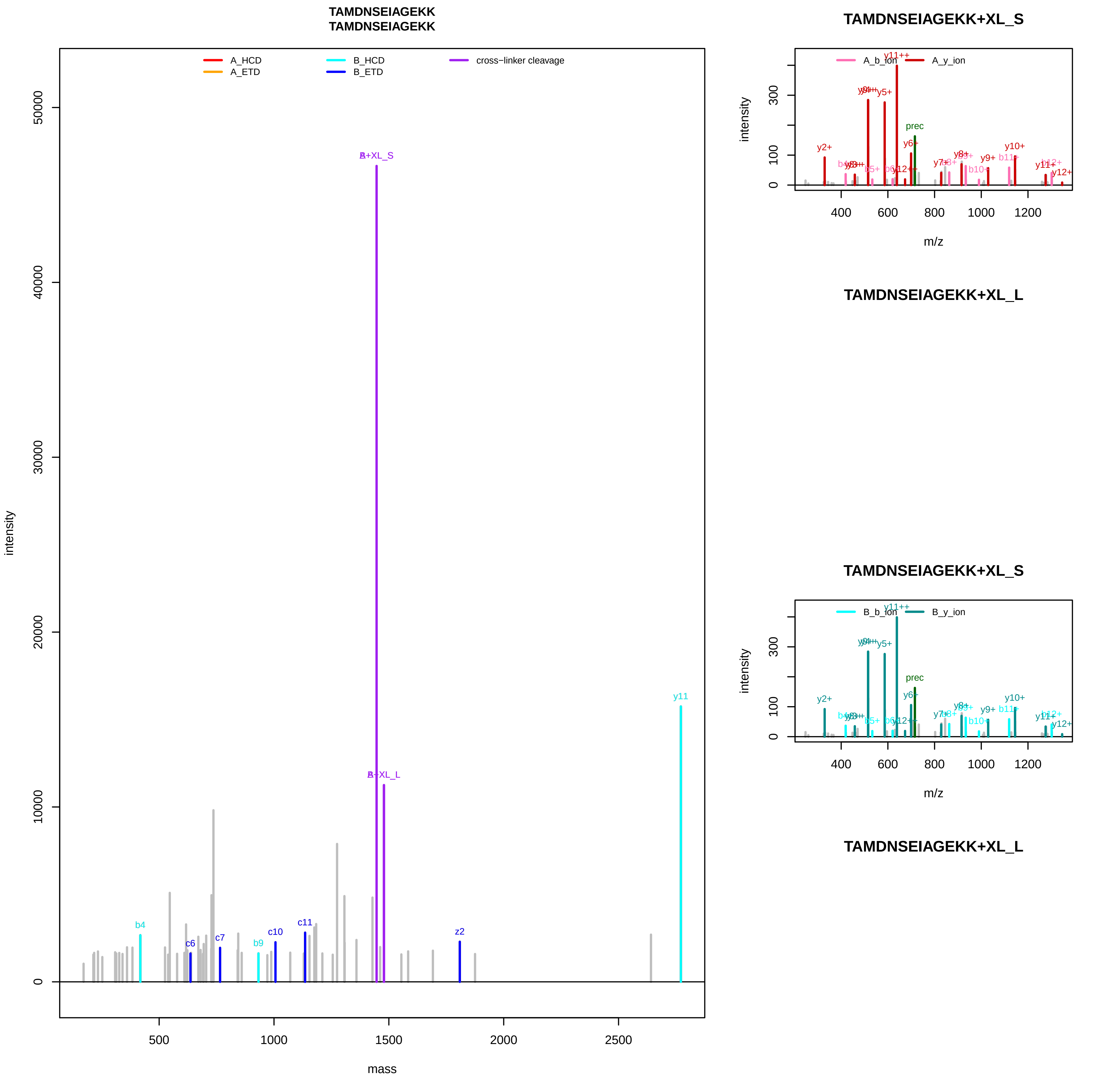
TAMDNSEIAGEKK
TAMDNSEIAGEKK
A_HCD
A_ETD
B_HCD
B_ETD
cross−linker cleavage
intensity
mass
A+XL_S
B+XL_L
b4
c6
c7
b9
c10
c11
z2
y11
0
10000
20000
30000
40000
50000
500
1000
1500
2000
2500
TAMDNSEIAGEKK+XL_S
A_b_ion
A_y_ion
y11++
y4++
y5+
prec
y2+
y6+
y8+
y7+
y9+
y10+
b11+
b10+
b12+
y11+
y12+
b5+
b6
y12++
intensity
m/z
0
100
300
400
600
800
1000
1200
TAMDNSEIAGEKK+XL_L
TAMDNSEIAGEKK+XL_S
B_b_ion
B_y_ion
TAMDNSEIAGEKK+XL_L
